# Supplementary material for: EnzML: multi-label prediction of enzyme classes using InterPro signatures
Source: BMC Bioinformatics. 2012 Apr 25;13:61. doi: 10.1186/1471-2105-13-61 (PMC3483700; doi:10.1186/1471-2105-13-61)
Supplement: Addtional file 5 — The Java code to format the data files, evaluate and predict. The file enzml_java_code.tar.gz contains the Java code used to format database data to ARFF and XML formats, to execute cross and train-test (jackknife) evaluations and to record evaluation results to database. More information is included in the readme.txt file and the Javadoc files. The code can be used with a MySQL database. To use a different database software, other JDBC drivers might be required. [file 1471-2105-13-61-S5.gz › java_code/utils/doc/edu/cornell/lassp/houle/RngPack/package-summary.html]

edu.cornell.lassp.houle.RngPack


---


|  |  |  |
| --- | --- | --- |
| **Overview** \| **Package** \| Class \| **Use** \| **Tree** \| **Deprecated** \| **Index** \| **Help** | |  |
| **PREV PACKAGE**   **NEXT PACKAGE** | **FRAMES**    **NO FRAMES**     **All Classes** |  |


---

## Package edu.cornell.lassp.houle.RngPack

| **Class Summary** | |
| --- | --- |
| **RandomApp** | RandomApp is a simple application that demonstrates the use of RngPack. |
| **RandomElement** | RandomElement is an abstract class that encapsulates uniform random number generators. |
| **RandomJava** | RandomJava is a class wrapper for the `Math.random()` generator that comes with Java. |
| **RandomSeedable** | `RandomSeedable` is an abstract class that extends the `RandomElement` class to include the ability to automatically generate a valid `long` seed from the clock. |
| **RandomShuffle** | RandomShuffle uses one random number generator to shuffle the numbers produced by another to obliterate sequential correlations. |
| **Ranecu** | Ranecu is an advanced multiplicative linear congruential random number generator with a period of aproximately 10^18^. |
| **Ranlux** | RANLUX is an advanced pseudo-random number generator based on the RCARRY algorithm proposed in 1991 by Marsaglia and Zaman. |
| **Ranmar** | RANMAR is a lagged Fibonacci generator proposed by Marsaglia and Zaman and is a good research grade generator. |

---


|  |  |  |
| --- | --- | --- |
| **Overview** \| **Package** \| Class \| **Use** \| **Tree** \| **Deprecated** \| **Index** \| **Help** | |  |
| **PREV PACKAGE**   **NEXT PACKAGE** | **FRAMES**    **NO FRAMES**     **All Classes** |  |


---
